# Ohio School for the Blind

**Published:** 1842-03

**Authors:** 


### OHIO SCHOOL FOR THE BLIND.

We have just received the Fifth Annual Report of this institution, and are happy to announce its increasing prosperity. Opening as late as the 4th of July, 1835, it has, already, 50 pupils, of which nearly all are the children of poor parents. But this number embraces a fraction only of those who from that State alone, to say nothing of the other States of the West, ought to be at this moment enjoying the advantages of such an institution. The neglect of parents, especially those of the country, in regard to the early treatment of the inflammations of their children's eyes, is truly lamentable; and the inefficiency of our plans of treatment, is too often quite as apparent. Hence the majority of the blind in the West, become so from diseases, which met in the outset with appropriate remedies, might be subdued. The number of this unfortunate class is annually increasing in all the Western States, none of which, except Ohio, has yet established a school. It is proper, then, that physicians should know, that many additional pupils can be received into the establishment at Columbus, and that the children of other States are not excluded. All the elementary branches of a common education are taught in the school; and also a number of mechanical arts. The expense of a pupil is $100 *per annum*, exclusive of clothing and some contingencies. The Report of Mr. Chapin, the excellent Superintendent, from which we have collected these facts, is drawn up with perspicuity; and has appended to it a number of interesting letters and other compositions, written by the scholars of the institution, which evince a progress as rapid as that made by children who have not lost their sight.

